# Risk Mitigation of Immunogenicity: A Key to Personalized Retinal Gene Therapy

**DOI:** 10.3390/ijms222312818

**Published:** 2021-11-26

**Authors:** Juliette Varin, Clément Morival, Noémien Maillard, Oumeya Adjali, Therese Cronin

**Affiliations:** CHU de Nantes, INSERM UMR1089, Translational Gene Therapy for Genetic Diseases, Université de Nantes, F-44200 Nantes, France; juliette.varin@univ-nantes.fr (J.V.); clement.morival@etu.univ-nantes.fr (C.M.); noemien.maillard@gmail.com (N.M.)

**Keywords:** immunogenicity, inflammation, gene therapy, AAV

## Abstract

Gene therapy (GT) for ocular disorders has advanced the most among adeno-associated virus (AAV)-mediated therapies, with one product already approved in the market. The bank of retinal gene mutations carefully compiled over 30 years, the small retinal surface that does not require high clinical vector stocks, and the relatively immune-privileged environment of the eye explain such success. However, adverse effects due to AAV-delivery, though rare in the retina have led to the interruption of clinical trials. Risk mitigation, as the key to safe and efficient GT, has become the focus of ‘bedside-back-to-bench’ studies. Herein, we overview the inflammatory adverse events described in retinal GT trials and analyze which components of the retinal immunological environment might be the most involved in these immune responses, with a focus on the innate immune system composed of microglial surveillance. We consider the factors that can influence inflammation in the retina after GT such as viral sensors in the retinal tissue and CpG content in promoters or transgene sequences. Finally, we consider options to reduce the immunological risk, including dose, modified capsids or exclusion criteria for clinical trials. A better understanding and mitigation of immune risk factors inducing host immunity in AAV-mediated retinal GT is the key to achieving safe and efficient GT.

## 1. Introduction

Recombinant adeno-associated virus (rAAV)-derived vectors have been the technological key enabling in vivo gene transfer in the last decade, with hundreds of clinical trials underway. This success is due to the efficacy as well as an excellent preclinical safety record of these vectors. However, as the number of clinical trials using rAAV has grown, so too have the number of reported adverse events. Preexisting and induced host immune responses to rAAV vectors have indeed emerged as a major limitation, undermining efficacy or even leading to a hold of some clinical trials [1,2,3]. More recently, innate immune responses elicited by AAV that cause explicit inflammation have started to garner attention, putting into question the impeccable safety record of the recombinant vector [4,5,6].

Ocular therapies have made the most significant progress in GT over the past 12 years and currently account for 30 active clinical trials. Such rapid progress is thanks in part to the immunomodulatory ocular environment that preserves the eye, in particular the retinal tissue, from a neutralizing immune reaction that could dampen transgene expression. Signs of neuroinflammation that have arisen in retinal GT trials may go unreported as the inflammation can generally be managed with steroid treatment. In the recent example of ADVM-022 clinical trial for diabetic macular edema (DME), timely and transparent reporting revealed significant inflammation in patients receiving the high dose of the rAAV vector, delivered through intravitreal injection. Five patients suffered from a rapid increase in ocular pressure refractory to conventional drug treatments, leading to surgery for three and to loss of vision for one patient. It is relevant for this review that DME patients have comorbidities, such as vascular disease, that may influence inflammatory factors [7].

**Figure 1:**
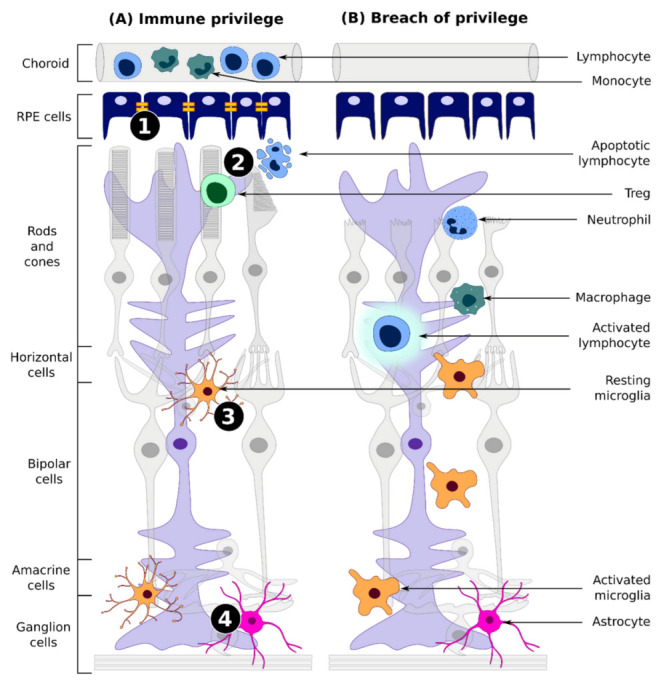
Retinal immune system. (**A**) In normal immunological conditions, multiple mechanisms are dedicated to immune tolerance and immune privilege. (1) The blood-retina barrier, which is partly composed by tight junctions (depicted in yellow) between RPE cells, acts as a physical and immune bulwark. It blocks the entry of circulating immune cells inside the retina and prevents antigens from escaping the retina [14,15,16]. (2) The retinal microenvironment, composed of immunoinhibitory and trophic factors secreted by the RPE, Müller glial cells, or astrocytes, promotes cell survival and tolerogenic immune responses (impairment of activated T-cell and APC functions, promotion of T-reg induction, and mitigation of inflammatory processes) [8,17,18,19,20,21,22]. (3) Microglial cells, often described as immune wardens, constitute the bulk of the retinal immune cells. They are found in a resting state in the healthy retina [23,24]. (4) Microglial cells are assisted by the complement system, a few resident macrophages and other retinal cells that perform immune surveillance and can act as APCs: Müller glia, astrocytes, or RPE cells [25,26]. While the immune tolerance mechanism tries to counterbalance inflammation, danger signals in the retina may still result in mild activation of the innate immune system, in a process recently termed para-inflammation [27,28,29]. (**B**) During stronger challenges, disruption of the immune privilege may occur. Recruitment and activation of circulating or resident immune cells in the retina, potentially including T cells, usually result in a full-fledged immune response and inflammation, causing permanent tissue damage [30,31].

The retinal immunological environment is protected from the overt immune reactions by mechanisms ranging from immune tolerance to immune privilege (IP). Among these mechanisms, we can cite a restricted transport of antigens and cells across the blood retinal barrier (BRB), a low level of major histocompatibility complex (MHC) on retinal cells, thus limiting antigen presentation, and the expression of a variety of immunosuppressive factors on ocular antigen-presenting cells (APC) [8]. An active local or peripheral tolerance to prevent runaway immune responses can be induced in some cases through the conversion of T cell to regulatory T cells (Treg) or the apoptosis of infiltrated effector T cells by inhibitory molecules expressed on the retinal pigmented epithelium (RPE) or resident stromal cells [9,10,11]. Nevertheless, this immune suppressive environment of the eye is only relative. Inflammation can be induced in some circumstances such as surgical trauma, patient history, disease physiopathology, or upon an escape of intracellular innate immune mechanisms to the immunosuppressive response of the eye [7] (Figure 1). 

In that case, T lymphocytes could become able to respond to ocular antigens, and even affect the retinal integrity through an effector cytotoxic response. A better understanding and monitoring of the mechanisms underlying this immunological escape is needed to reduce inflammation and increase the efficacy of rAAV treatment. Indeed, the increasing recent reports of neuroinflammation following ocular rAAV-mediated GT could justify retina-specific immunomonitoring criteria or adjuvant therapy such as hydroxychloroquine as proposed by Chandler et al. for retinal rAAV treatments [12]. Furthermore, less immunogenic vectors should be prioritized such as that designed by Chan et al. utilizing TLR9 inhibitory oligonucleotides to avoid immune activation [13]. In this review, we consider the immunogenic responses that may circumvent the IP of the retina and may require close attention in order to establish safe and personalized ocular gene transfer. In particular, we review innate immune key actors, and more specifically, microglial responses that may be evoked by the immunogenic sequences carried in many therapeutic vectors. We also consider strategies to mitigate the immunological risk of rAAV-based ocular new treatments.

## 2. Immune Responses in Retinal Gene Therapy Trials

The occurrence of immune responses following rAAV ocular delivery has arisen with the increasing number of clinical trials. Table 1 presents a non-exhaustive list of immune responses reported in patients and published to date. Note that others have published thorough recent reviews on the status of such trials [32,33,34]. In most cases, no serious adverse events related directly to the study agents have been reported, supporting the remarkable safety of rAAV retinal GT. As shown in Table 1, activation of innate and adaptive responses at doses above 1 × 10^11^ vg is frequent, though humoral antibody responses to the transgene products remain rare. This is true for the subretinal injection (SRI) as well as intravitreal (IVT) route of injection, with the latter considered a more immunogenic route [5,35]. Frequently described is a post-operative elevation of intraocular pressure (IOP) and in most cases, inflammation arises concomitantly and is attributed to the invasive surgical procedure. In more serious events, vitreous cells may accumulate near the fovea, leading to a drop in visual acuity and warranting a more prolonged course of corticosteroids [36].

However, there are reasons to suspect non-surgical triggers of inflammation. The inflammation described in dose-escalation studies reveals the higher titer cohort to be most at risk. This dose-dependence supports a vector-related pro-inflammatory immune response. Moreover, inflammation often arises 2–3 weeks after treatment when surgical trauma should have resolved. It may be considered unnecessary to untangle the source of the late-onset inflammation when it is responsive to a steroid treatment with few side effects. However, some adverse events linked to the glucocorticoids themselves have already been described in retinal rAAV trials which point out the need for other alternatives to mitigate the inflammation [37]. Furthermore, there is a low risk of infection following the subretinal surgery, in which case immunosuppression is not ideal. In conclusion, all these reports point out the limits to the immunosuppressive and anti-inflammatory ocular processes that protect the retinal tissue and its poor cellular renewal capacity from the transient effects of inflammation. The ocular immune privilege is only relative and it is mandatory to better understand the physiological aspects leading to a break of the retinal immune suppressive environment in particular settings of rAAV delivery.

**Table 1:** Retinal GT trials involving rAAV vectors and summary of immune responses. P: Phase, NR: non-randomized, R: randomized, OL: open-label, M: masked.

NCT Number	Sponsor	Study Type (1)	Vector					Immune Response		References
			Serotype	Promoter	Transgene	Dose (vg)	Injection (2)	Inflammation	Other	
**Leber congenital amaurosis**										
NCT00516477	Spark Therapeutics	PI, NR, OL	2	CBA	*hRPE65v2*	1.5 × 10^10^ to 1.5 × 10^11^	SRI	no inflammation	transient increase in nAB in 2/3 patients	Maguire et al. 2009 [38] Simonelli et al. 2010 [39]
NCT01208389		PI/II, NR, OL (follow-on study: injection of the contralateral eye)				1.5 × 10^11^	SRI	no inflammation	transient minor increase in anti-capsid Abs 1/10 no cell-mediated T cell responses detectable in peripheral blood	Bennett et al. 2012 & 2016 [40,41]
NCT00999609		PIII, R, OL				1.5 × 10^11^	SRI	2/20 with transient mild bilateral inflammation in treated arm	N/A	Russell et al. 2017 [42]
NCT00643747	University College, London	PI/II, NR, OL	2	hRPE65p	*hRPE65*	1 × 10^12^	SRI	in 5/8 with high dose; anterior uveitis in 1/8	increased level of AAV2 NAbs and marginal increased circulating T cells with reactivity to AAV2 in 1 high-dose patient transiently increased circulating neutralizing antibodies to AAV2 in another high-dose patient	Bainbridge et al. 2015 [37]
NCT01496040	Nantes University Hospital	PI/II, NR, OL	2	hRPE65p	*hRPE65*	1.22 × 10^10^ to 4.8 × 10^10^	SRI	transient infra-clinical inflammation at D+4 in three patients more significant transient inflammation was observed in two patients	anti-AAV4 IgG antibodies in three patients (1 before + 2 after injection) weak and transient cellular response to RPE65 between	Le Meur et al. 2018 [43]
NCT02781480	MeiraGTx UK II Ltd.	PI/II, NR, OL	5	hRPE65p^opt^	*hRPE65^opt^*	1 × 10^11^ to 1 × 10^12^	SRI	mild uveitis in 3/9 low, 1/3 intermediate, and 1/3 high dose serious uveitis in 2/3 intermediate dose and 1/3 high dose	N/A	clinicaltrials.gov 24 November 2021
NCT00481546	University of Pennsylvania	PI, NR, OL	2	CBSB	*hRPE65*	5.96 × 10^10^ to 1.79 × 10^11^	SRI	N/A	episodic humoral immune response in 4/15; modest increase in T-cell response by cultured ELISpot in 3/15	Jacobson et al. 2012 [44]
NCT00749957	Applied Genetic Technologies Corp	PI/II, NR, OL	2	CBSB	*hRPE65*	1.8 × 10^11^ to 6 × 10^11^	SRI	in 3 patients, eye inflammation at highest dose	Titers of neutralizing antibodies to AAV increased in 5 of 12 patients	Weleber et al. 2016 [45]
NCT00821340	Hadassah Medical Organization	PI	2	N/A	*hRPE65*	N/A	SRI	N/A	N/A	
**Choroideremia**										
NCT02341807	Spark Therapeutics	PI/II, NR, OL	2	CAG	*hCHM*	5 × 10^10^ to 1 × 10^11^	SRI	N/A	N/A	Morgan et al. 2021 [46]
NCT01461213	University of Oxford	PI/II, NR, OL	2	CAG	*hCHM* + *WPRE*	0.6 × 10^10^ to 1 × 10^11^	SRI	vector-related inflammation and vitritis in 1/14 at 2wpi;	N/A	MacLaren et al. 2014 [47]
NCT02077361	Ian M. MacDonald	PI/II, NR, OL				1 × 10^11^	SRI	1/6 severe intraretinal inflammation, leading to permanent structural and functional impairment of the retina	N/A	Dimopoulos et al. 2018 [48]
NCT02553135	Byron Lam	PII, NR, OL				1 × 10^11^	SRI	1/6 vitreous cells	significantly increased serum anti-AAV-2 neutralizing antibody after treatment 1/6	Lam et al. 2019 [49]
NCT02671539	STZ eye trial	PII, R, OL	2	CAG	*hCHM* + *WPRE*	1 × 10^11^	SRI	N/A	N/A	Fischer et al. 2019 [50]
NCT02407678	University of Oxford	PII, R, OL				1 × 10^11^	SRI	N/A	N/A	
NCT03507686	NightstaRx Ltd.	PII, NR, OL				N/A	SRI	N/A	N/A	
NCT03496012	NightstaRx Ltd.	PIII, R, M				N/A	SRI	N/A	N/A	

**X-linked retinitis pigmentosa**										
NCT03116113	NightstaRx Ltd.	PI/II, R, M	8	hRK	*coRPGR*	2 × 10^9^ to 4 × 10^11^	SRI	subretinal inflammation at high doses	N/A	Cehajic-Kapetanovic et al. 2020 [36]
NCT03252847	MeiraGTx UK II Ltd.	PI/II, R, OL	5	hRK	*RPGR*		SRI	Inflammatory responses were observed in 2 out of the 3 patients in the high-dose cohort	N/A	Michaelides et al. 2020 [51]
**Retinitis pigmentosa**										
NCT04919473	Nanoscope Therapeutics Inc.	PI/iIa, NR, OL	2	CMVp + mGLUR6 enhancer	*MCO*	1.75 × 10^11^ to 3.5 × 10^11^	IVT	N/A	N/A	clinicaltrials.gov 24 November 2021
**Age-related macular degeneration**										
NCT01494805	Lions Eye Institute, Perth	PI/II (dose-escalation), R, M	2	CBA	*sFLT1*	1 × 10^11^	SRI	2 anterior chamber inflammation and 1 eye inflammation; two ocular aEs were considered possibly related to rAAV. sFLT-1 was eye inflammation, and anterior chamber inflammation, which was mild in nature and resolved without sequelae	IFNϒ T-cells against capsid in 1/21; 3 seroconverted;	Constable et al. 2016 [52]
NCT03066258	Regenxbio Inc.	PI/iIa (dose-escalation), NR, OL	8	N/A	soluble anti-VEGF (monoclonal antibody fragment)	3 × 10^9^ to 2.5 × 10^11^	SRI	post-operative inflammation in 36% of subjects resolved within days to weeks	N/A	Allen Ho et al. 2021 [53] (presentation)
NCT01024998	Genzyme	PI (dose-escalation), NR, OL	2	CBA	*sFLT1*	2 × 10^8^ to 2 × 10^10^	IVT	2/3 intraocular inflammation resolved with topical steroid	62% of patients injected with 6 × 109 or 2 × 1010 had increase in anti-AAV2 antibodies	Heier et al. 2017 [54]
NCT03748784	Adverum Biotechnologies, Inc.	PI, NR, OL	2.7m8	CMV	aflibercept	2 × 10^11^ to 6 × 10^11^	IVT	Ocular inflammation minimal and responsive to steroids in low-dose cohort	N/A	Adverum press release 01 October 2021 [55]
NCT03585556	Janssen Research & Development, LLC	PI, NR, OL	2	CAG	*sCD59*	3.56 × 10^11^ to 1.071 × 10^12^	IVT	N/A	N/A	
NCT03144999	Janssen Research & Development, LLC	PI, NR, OL	2	CAG	*sCD59*		IVT	N/A	N/A	
**Diabetic macular edema**										
NCT04418427	Adverum Biotechnologies, Inc.	PII, R, OL	2.7m8	CMV	aflibercept	2 × 10^11^ to 6 × 10^11^	IVT	5/12 increase ocular pressure and 1/12 loss of vision at high dose	N/A	Adverum press release 22 July 2021 [56]
**X-linked retinoschisis**										
NCT02416622	Applied Genetic Technologies Corp	PI/II, NR, OL	2tYF	CBSB	*hRS1*	1 × 10^11^ to 6 × 10^11^	IVT	10/21 anterior chamber cells for intermediate and high doses	N/A	clinicaltrials.gov 24 November 2021
**Leber hereditary optic neuropathy**										
NCT02161380	Byron Lam	PI	scAAV2	CAG	ND4 subunit gene of complex I with targeting sequence of P1 isoform of subunit c of ATP synthase	5 × 10^9^ to 2.46 × 10^10^	IVT	1/14 mild uveitis medium dose and 1/14 uveitis low dose after 2 months, resolved spontaneously	1/14 strong increase in NAbs	Guy et al. 2017 [57]

## 3. The Immunological Environment of the Retina

### 3.1. Immune Privilege

Immune privilege in the eye is composed of a barrier function and a physiological function. The purpose is to first block any foreign antigen that may activate an immune response and, should that fail, to diffuse the immune reaction with the overriding goal to limit neuro-retinal loss. In extreme cases, this may lead to supporting a latent persistent infection. The first line of protection comes from endothelial, epithelial, and glial barriers (reviewed by Forrester et al. 2018 [58]). At the epithelial-CNS junction lays the blood-aqueous barrier (BAB) of the eye, while at the blood-CNS junction is the blood retinal barrier (BRB). The BRB comprises a double-layered epithelial barrier continuous with the retinal pigment epithelial cells (RPE) and the vascular endothelial cells of the iris and Schlemm’s canal. The BRB function depends on the tight junctions connecting endothelial cells of retinal vessels to each other and the RPE cells similarly. These tight junctions limit the selective diffusion of ions and small solutes between neighboring cells, while receptors homeostatically regulate the CNS transport of smaller molecules and block the entry of infiltrating cells and viruses. Some viral proteins, such as HIV-gp120, are shown to disrupt BRB permeability by directly downregulating the expression of tight junction proteins [59].

When a barrier is breached, physiological aspects of immune privilege come into play, coordinating responses to achieve suppression and/or tolerance of the pathogen. The anterior chamber, vitreous cavity, and subretinal space of the retina are largely exempt from systemic immune surveillance with the resident immune cell, the microglial cell, expressing low levels of pathogen recognition receptor (PRRs) in resting state [60]. Moreover, immunosuppressor proteins can be released by neurons and microglia in the presence of an antigen, such as a virally delivered transgene product. The immunosuppressor proteins, including CX3CL1 and CD47 (to control microglial and macrophage function) and CFH (to control T-cell activation and block complement triggers), are already activated by neurodegeneration and block the overproduction of pro-inflammatory signals (such as inducible nitric oxide synthase, interleukin (IL)-1β, tumor necrosis factor-α (TNF-α), and IL-6) [61,62]. Indicative of the importance of this immunosuppressor function is the recent proof that the minor haplotype of chromosome10q26, a strong risk factor for the development of age-related macular degeneration (AMD), results in inhibition of CD47-signalling leading to chronic subretinal inflammation [63]. Still, little information is available on the involvement of effector T cells in retinal degenerations. Elevated levels of effector T cell IL22 and IL17 cytokines were reported in AMD patients [64,65], suggesting an activation of cellular-mediated immunity. The ability of T cells to activate and respond to retinal antigens in inflammatory settings has become increasingly evident in the last decade. As the resulting cytotoxic response may affect the integrity of the eye, it is critical to better characterize and predict those responses in pathological settings or after the delivery of an ocular treatment.

Inflammatory immune responses for ocular gene therapies become harder to predict in the clinical setting, as both physiological and barrier functions of immune privilege may already be compromised by aging or the ocular disorder physiopathological conditions such as inflammation or oxidative stress induction [21,66,67]. Indeed, it is clear from the first generation of GT products under clinical trial, that disease status plays a major role in the likelihood of the induction of an innate neuroinflammatory response. However, other more controllable factors also play a role, in particular the dose of AAV, the serotype, and the route of delivery. In retinal preclinical and clinical trials, neuroinflammation can arise at 2 weeks post-injection, suggesting the immunosuppressive, anti-inflammatory ocular environment is being overcome by the innate response. The “immune sentinel” of the retina, the resting microglia, determines the nature of the innate immune response in these spaces and is the key checkpoint between limiting or aggravating neuroinflammation [66].

### 3.2. Microglial-Based Surveillance and Its Impact on AAV Gene Therapy

**Figure 2:**
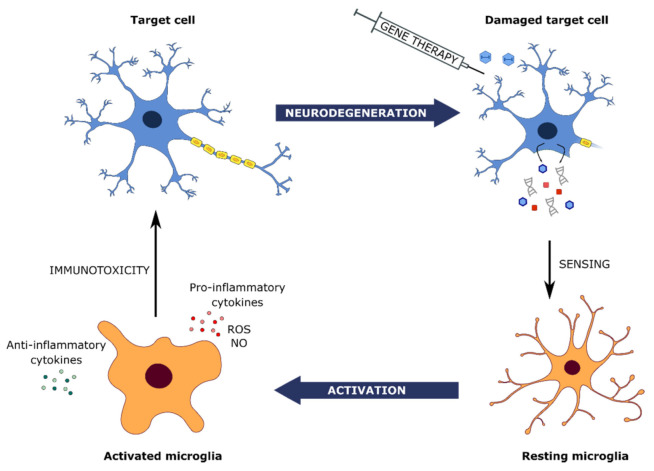
The central role of microglial cells in the viral vector sensing and induced immune toxicity. Activation of microglial cells after vector viral sensing can lead to pro-inflammatory cytokine release, promoting retinal cell or neuronal death in the context of endangered cells. In turn, cell degeneration may favor microglial activation. ROS: reactive oxygen species; NO: nitric oxide.

Described for the first time by del Rio-Hortega in 1919, these macrophage-like cells exert many different functions, ranging from homeostasis to immune defense. In physiological conditions, microglia can be found displaying a ramified morphology, associated with its “resting” state [24] (Figure 2). Resting may be a misleading term, as these cells continually monitor their environment, and are constantly moving [67]. In fact, they express a wide variety of proteins capable of sensing their surroundings, referred to as “sensome”, and have a wide dynamic range of expression for the host of receptors that respond to the environment [24]. In addition to receptors for neurotransmitters, chemokines, and cytokines, the microglia express PRRs, sensors of innate immunity. PRRs recognize pathogen non-specific molecular motifs such as lipopolysaccharides (LPS) or nucleic motifs.

In response to injury or infection, homeostatic microglial cells are able to shift into different functional states, becoming “activated”, as can be observed in their morphological changes, exerting an amoeboid shape [24]. The activation process of microglia varies heavily, depending on the stimuli, and is regulated by several intercellular interactions involving cell-surface molecules and soluble mediators, such as cytokines, ROS, and neurotransmitters [68]. Intercellular interactions that regulate microglial activation involve cross-talk of microglia with neurons, with the blood–brain barrier (BBB), with astrocytes, and with T-cells which infiltrate the retina or CNS parenchyma [69].

Traditionally, macrophage and microglia activation has been classified in two different states: classic (M1) state, considered as pro-inflammatory; and alternative (M2) state, considered as anti-inflammatory. However, this classification does not quite correspond to the variety of microglia phenotypes, and has been a subject of discussion in the last decade [70]. Lineage tracing and transcriptomic studies have led to deeper understanding of the microglial activation states (reviewed by Chen et al. 2020; Mathys et al. 2017 [71,72]). Single cell RNA sequencing has been particularly useful, with at least 9 distinct microglial signatures identified with varying predominance, depending on the health status of the neuronal tissue [73,74]. 

Microglial cells participate in innate immune response in two major ways. First, they stimulate neuroinflammation, secreting two main cytokines: tumor necrosis factor (TNF)-α and interleukin (IL)-1β, as well as other interleukins [75]. Second, they recognize and phagocytose apoptotic neurons and retinal cells, debris, and microorganisms. The efficient removal of dying cells is considered an overall anti-inflammatory mechanism, as it prevents further release of cytokines and chemoattractants [76]. However, during diseases or inflammatory challenges, microglia have been shown to phagocytize endangered or damaged but still viable cells, playing a role in accelerating degeneration [77]. This can be aggravated by rAAV-mediated gene therapies, where phagocytosis of the debris from rAAV-transfected cells may lead to activation of the phagocytosing and neighboring microglia, due to viral DNA present in the phagocytosed debris. Viral DNA recognition by PRRs can generate an auto-activating loop that unleashes increased activated microglial activity in the transduced tissue. In most therapeutic contexts, rAAV-mediated expression of the therapeutic transgene may supply a lost function but may not stall degeneration. In an already inflamed or damaged tissue, activation of microglia may lead to further cytotoxic effects, neglecting the positive outcome of therapies.

### 3.3. DNA Sensors

**Figure 3:**
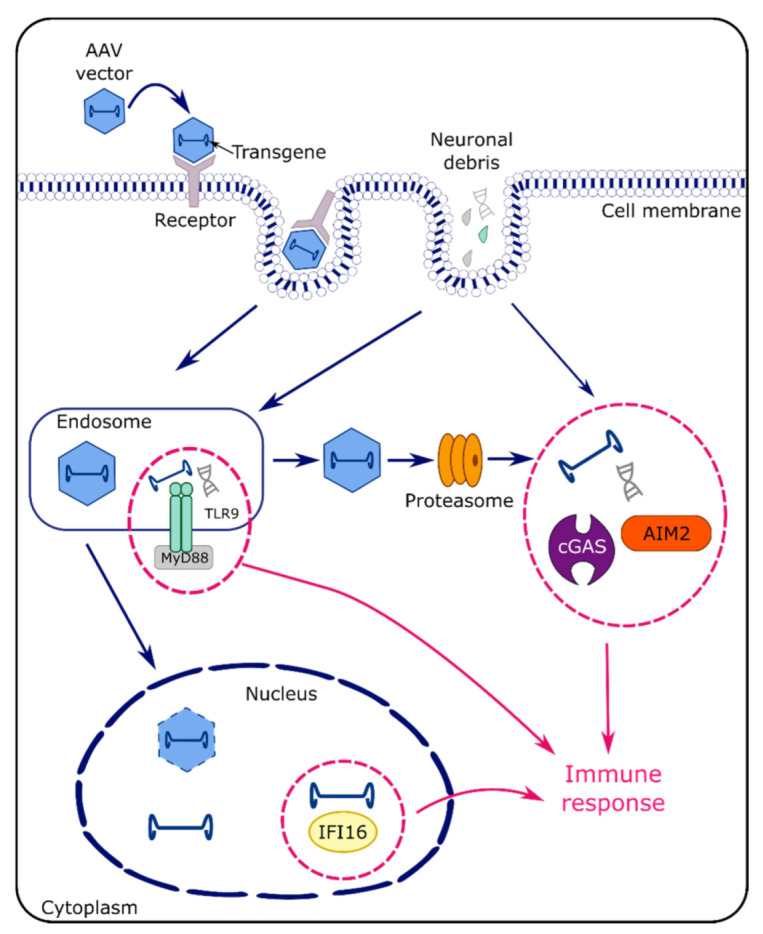
Microglia-mediated immune response may be triggered by DNA sensing of therapeutic DNA sequences. Viral vector sensing can occur in diverse compartments of the cell, following endocytosis of therapeutic rAAV particles or phagocytosis of transduced cell debris containing DNA. TLR9 recognizes CpG hypomethylated DNA sequences in the endosome, AIM2, and cGAS recognize dsDNA in the cytosol, and IFI16 recognizes dsDNA mainly in the nucleus. Red dashed circles depict possible DNA sensing. RPE: retinal pigmented epithelium; HC: horizontal cells; BC: bipolar cells; AC: amacrine cells; GC: ganglion cells.

DNA sensors are a subtype of PRRs, playing a key role in innate immunity. These sensors are able to detect DNA derived from microbial infections or aberrant self-DNA, and are associated with adaptor proteins capable of triggering immune responses. Multiple sensors have been described, depending on their subcellular localization, their specificity towards nucleic acids, as well as their associated adaptor protein, including cGAS, AIM2, IFI16, and TLR9 [78] (Figure 3).

Two sensors capable of recognizing double-stranded (ds)DNA and which are found, crucially, in the cytosol are cyclic GMP–AMP synthase (cGAS), an enzyme associated with downstream adaptor molecule stimulator of interferon (STING), and absent in melanoma 2 (AIM2), whose activation leads to the assembly of a multi-protein complex stimulating inflammatory caspases called the inflammasome [79,80]. Interferon (IFN)-γ inducible protein 16 (IFI16) is another DNA sensor from the same family as AIM2, which can be found predominantly in the nucleus, with smaller quantities in the cytoplasm. Also capable of recognizing dsDNA, IFI16 is linked with STING adaptor and is involved in inflammasome formation [78,81]. Finally, toll-like receptor (TLR)-9, member of the well-studied TLR sub-family of PRRs, is the only DNA sensor expressed in the endosome and the only one, to date, experimentally proven to trigger rAAV-associated immunity. TLR9 recognizes unmethylated CpG motifs from single-stranded (ss)DNA or dsDNA, and is teamed with adaptor protein myeloid differentiation primary response gene 88 (MyD88). TLR9 activation induces the assembly of a supramolecular organizing center called the myddosome, involving MyD88 and multiple members of the serine threonine kinase family IRAK [82,83]. Subsequent signaling leads to induction of pro-inflammatory cytokines, including TNFα, IL-1β, IL-6, IL-12, IFN-α, and IFN-λ [78,83,84,85,86].

### 3.4. The TLR9/MyD88 Pathway Is Activated upon rAAV Transduction

To experimentally investigate TLR9-MyD88 pathway, TLR9 activation can be elicited by using natural nucleic acids (mainly viral vectors) or synthetic oligodeoxy nucleotides (ODN), termed CpG ODNs. CpG ODNs are listed in three main classes, depending on their characteristics. Class A is the closest to the natural CpG found in viral or bacterial DNA; it activates mainly NK cells and plasmacytoid DC precursors leading to IFNα expression. Class B has primarily a B cell stimulatory effect and has less in common with natural CpG ODNs and finally, class C has combined features of class A and class B [87]. Topical application of CpG ODNs onto a scratched cornea was shown by Chinnery et al. to lead to inflammation in eye tissues including retina, after CpG leakage into the anterior segment [88]. Deguine and Barton have shown that the nature of the TLR9 response−IFN type I or pro-inflammatory cytokine production is dependent on the class of CpG motif (class A or B) and the composition of phospholipids in the membrane incorporating TLR9. It has also been demonstrated that the pro-inflammatory cytokine response requires a localization motif in the tail of TLR9 while the anti-inflammatory IFN type I response, by contrast, requires the AP-3 adaptor [89].

The TLR9 pathway has been of interest in the GT community due to the potential implications for viral-vector-mediated gene transfer. Since inflammation following injection of rAAV has been repeatedly observed, the TLR9-mediated activation of the innate response should be considered a risk factor for retinal rAAV GT. The question currently being posed by many in GT is how relevant is this risk? Does it warrant the significant attention it has received in the past few years? In particular, it is worth considering that too much attention on TLR9 may result in the neglect of other DNA sensors that can also signal danger from rAAV, such as the already discussed IFI16, AIM2, and cGAS, or others like DHX10 and hnRNPA2. This notwithstanding, the bulk of evidence thus far points to TLR9 as a key culprit in the onset of immune signaling in response to rAAV genomes [90]. This has been the case since Martino et al. originally noted that TLR9-dependent innate immune signaling was induced by the self-complementary rAAV genome [91]. As described above, the innate immune response in the retina hinges on the activation state of microglia. Recent studies have shown that while no transgene expression was observed, viral particles can enter the immune cells such as dendritic and microglial cells; though the question remains open as to whether they can reach the nucleus or whether their presence is only detected at the endoplasmic reticulum [92,93].

Even though inefficient intracellular trafficking of vectors seems to prevent transgene expression, sensing of vector DNA may still happen and these cells may elicit TLR9-mediated immune responses. In particular, the low-pH-triggered structural and autoproteolytic changes to the rAAV capsid necessary for endosome escape, may lead to premature virus uncoating and exposure of the triggering vector DNA. However, specific transduction of the microglia by rAAV is not necessary for microglia to be exposed to transgene DNA and ITR sequences as these cells have phagocytic properties. As GT in the retina and CNS is often held in neurodegenerative contexts, it is also possible for the transduced cell to degenerate due to natural disease progression and for the resulting cellular debris to be phagocytosed by microglial cells. Debris still carrying vector sequences may as a consequence also be able to induce anti-vector immune responses. As such, strategies aiming at detargeting those cell sensors from rAAV transduction may not be sufficient to dampen vector recognition and host immunity.

## 4. Factors Influencing Inflammatory Reactions in Response to rAAV

### 4.1. CpG Content

The type of vector sequences that could initiate a destructive immune reaction in the retina have been examined by Xiong et al. [94]. They propose that associated cis-regulator elements pose a major risk with CMV and CAG promoters which potentially trigger degeneration of retinal cells. UbiC and Best1 promoters as well were described in this study as being detrimental for RPE cells. A number of hypotheses are proposed by this group to account for such an effect, amongst them TLR9 activation of an immune response due to viral-like signatures present within the promoters.

Table 2 examines sequences used in therapeutic rAAV vectors, including those referenced in Table 1. The risk factor 1 (RF1) is calculated as previously described [95]. This table does not show the direct risk of TLR9 activation, which is likely dependent on CpG-DNA secondary structure, but it demonstrates the risk relative to the number of CpG sequences. Although GTCGTT has been proposed as an optimal human TLR9 activating sequence, other sequences of CpG ODN are also activating and questions remain regarding the potency of activation through sequence context, TLR secondary structure, and cell type [96,97,98].

### 4.2. Combination of Viral and Bacterial Sequences

One source of potential inflammatory ITR/transgene combinations is the new generation of gene therapies involving microbial base editors and Cas9 nucleases to achieve gene editing. To date, toxicity found in vivo using Cas9 has frequently been attributed to off-target effects; however, the Cas endonucleases are bacterial-derived and carry a relatively high frequency of unmethylated CpG motifs compared to therapeutic genes currently in clinical trials (Table 2). As such, they may be more susceptible to engaging TLR9 activation and to generating inflammation. To avoid this, first generation Cas9 proteins can be modified to carry 5-methylcytosine, analogous to the well-known molecular defense strategy of phage T4 in adding hydroxymethyl groups to its cytosines [99]. However, it is possible that even with modifications, the combination of bacterial sequence within a rAAV cassette may pose a particular risk. It is especially the case with an ITR secondary structure next to a bacterial sequence enriched in CpG, compounding the activation of multiple PRRs. Delivering nuclease as a ribonucleoprotein (RNP) or as an mRNA seems more ideal, given that only transient expression of the nuclease is generally required. However, the challenge of transducing neuronal and retinal cells without a suitable vector remains a significant obstacle. Fortunately, thus far, there are no published reports describing negative effects of rAAV-encapsidated Cas9 and Editas Medicine has sponsored the first human clinical trial (NCT03872479) using Cas9 to target the eye. This trial is designed to treat photoreceptor degeneration in LCA10 caused by CEP290 mutations using a targeted AAV5-spCas9 [100]. As reported by Editas, patients receiving the high dose did not display concerning events up to 15 months after injection [101].

### 4.3. Route of Administration

As mentioned before, the injection ocular surgery influences the inflammatory responses. This has already been studied in animal models such as rodents, dogs, or non-human primates (NHP) but also more recently in patients. Reichel and colleagues demonstrated that NHP and patients did not develop anti-AAV8 antibodies following subretinal injection (SRI), while NHP receiving an intravitreal injection (IVT) had substantial humoral immune responses [5,102]. The same observations were made regarding AAV2 and AAV5 [103,104]. As one might expect, the route of administration also influences the biodistribution of the viral vector. Little or no extraocular distribution is found following a subretinal injection while the intravitreal route can lead to a more important systemic dissemination as shown by Seitz et al. 2017 [35]. Furthermore, as alluded to above, the immunosuppressive functions of the ocular immune privilege in the subretinal space may be induced by retinal degeneration, as well as the presence of antigen in vectored therapies delivered by a subretinal injection. The intravitreal injection of vector appears to lead to a less muted immune response, possibly more comparable to that observed in other parts of the CNS. This difference between SRI and IVT injection is often explained by the presence of macrophage in the vitreous humor; however, it is also worth noting that upon IVT injection, the retina is pierced, whereas it remains intact with SRI; the physical breaking of blood tissue barriers may then induce a systemic immune response [105].

### 4.4. Inflammatory Components of Retinal Diseases

The relative success of a retinal GT may be heavily influenced by the immune status of the patient. Inflammation is known to play an important role in the pathogenesis of several retinal disorders that would be candidates for GT. This is clearly the case for age-related macular degeneration (AMD), diabetic macular edema (DME), but also for some forms of the inherited retinal disorder retinitis pigmentosa (RP). In AMD, the oxidative stress may elicit a response also known as “para-inflammation” where the innate immune system induces a mild inflammatory response to restore homeostasis that, if chronic, may contribute to AMD through imbalanced inflammatory response [28]. Drusen, the hallmark of AMD, may be caused by RPE cell injury and their release of cyto- and chemokines to recruit dendritic cells, which finally amplify the inflammatory phenomenon and install chronic inflammation [106]. Complement activation playing a major role in AMD pathogenesis is yet another source of inflammation [107,108,109,110]. In DME, chronic hyperglycemia generates metabolic changes resulting in an increase in inflammatory cyto- and chemokines such as interleukin 6 (IL-6), IL-8, TNFα, or ICAM-1 which might alter vascular permeability and disrupt the blood-ocular barrier [7]. Finally, in RP, inflammation of the vitreous cells corresponds to a worse clinical picture with high levels of MCP-1, a pro-inflammatory marker known to activate microglial cells and recruit dendritic cells, monocytes, and memory T cells, identified in the vitreous body of RP patients [111]. All these studies show that regardless of the underlying pathology, retinal disorders invariably result in inflammatory tissue. It is unsurprising then that varied idiosyncratic immune responses can result when a viral vector is introduced to such a tissue.

## 5. Options to Reduce Immunological Risk

### 5.1. At the AAV Level

#### 5.1.1. Modified Capsid

Wild-type AAV are parvoviruses commonly encountered by humans, leading to a preexisting humoral and cellular immunity that is a major concern following rAAV delivery in GT protocols. In order to bypass these issues and a potential sensing of these known viral antigens in humans, there is a need for engineered rAAV capsids capable of evading the immune system [112]. Engineered vectors have arisen in the past years through the use of different approaches. For instance, the directed evolution-based vector engineering allows the testing of a large variety of rAAV variants in vivo, leading to the selection of the most specific for the tissue of interest [113]. Recently, Pavlou and co-workers published the identification of new AAV variants using the same approach. They identified several rAAV variants after inducing a shift in the geometry of the loop 4, which is also involved in the targeting of the capsid by neutralizing antibodies. They showed that these new variants have the ability to cross barriers, have a lower heparin affinity, have a capsid that is easier to disassemble, and are less sensitive to neutralization [114]. These variants led to an expression of the transgene in the canine retina, in the NHP fovea, and in human retinal explants, and restored function in a mouse model of achromatopsia [114]. Another vector selection strategy mines the parvovirus ancestral genome to identify capsid sequences that have coevolved with humans [115]. This strategy produced the Anc80 serotype with the promise of a safer vector for GT [116]. In addition to their proven escape of neutralization, all these new rAAV variants with their modified structure and capsid assembly may trigger in a different manner the viral sensing pathways described above. Chemical modifications of the capsid can also impact the interaction of the AAV with host immunity as recently shown by Mével and his team. In their recent issue, they describe a novel method to chemically graft N-acetylgalactosamine (GalNAc) on lysine residues present on the surface of AAV2 capsid. In vitro, using mouse hepatocytes, they showed increased transgene expression using chemically modified capsid compared to native AAV2 capsid. While in vivo data did not match the previous observation, neutralizing antibodies induction was greatly reduced in chemically modified capsid compared to standard capsid [117]. These new rAAV vectors have modified physical properties possibly modulating their intracellular trafficking which may have an impact on their recognition by the innate immune system. In conclusion, the sensing of new generation rAAV vectors with the innate system should be addressed in the subsequent studies and may offer a solution to escape or at least decrease vector recognition.

#### 5.1.2. Evading DNA Sensing

In addition to long-sought efforts to make the coat safer and modify viral vector whole structure, there are now strategies to make the DNA cargo safer. One approach relies on the incorporation of short DNA oligonucleotides into the vector genome, which antagonizes TLR9 as described by Chan et al. 2021. These engineered vectors appeared to be inherently less immunogenic and elicited strongly decreasing innate and T cell responses to the vector along with enhanced transgene expression [13].

Faust et al. used the immunogenic rAAVrh32.33 capsid to encapsidate a CpG-dimer depleted genome [118]. They found that the immunogenicity could be mitigated by the state of the genome. Mouse muscle transduced with rAAVrh32.33 carrying the CpG negative genome had reduced CD4+, CD8+, and MHC II expression compared to the unmodified vector and showed stable transgene expression. This was among the earliest demonstrations that the CpG content of the viral genome could impact the extent to which TLR9 is activated and, in turn, the extent to which the capsid antigens may provoke an adaptive response. Since this first proof of principle, Bertolini et al. have described the effect of CpG-tetramer depleted vector expressing hFIX transgene in a hemophilia B mouse model [119]. They found a substantial reduction in CD8+ T cell infiltration at 4 weeks post-treatment; however, they found only weak impact on the antibody titers raised against the capsid and the FIX transgene product. These depleted vectors may be capable of mitigating an innate immune response but stop short of reducing an adaptive response. The secondary structure of CpG stretches rather than the sequence itself may be a better predictor of the immune risk a genome may pose. Inhibitory oligonucleotides with stem-loop secondary structures have recently been used to antagonize TLR9 activation [13]. Using this deimmunized vector, they were able to show that intraocular inflammation following rAAV subretinal injection of 4 × 10^11^ vg per eye was mitigated. This inflammation was only delayed, however, suggesting that other viral DNA sensors may take the role of TLR9 when inhibited. Ultimately the value of such deimmunized vectors for neuronal and retinal GT will depend on the extent to which the innate immune responses are engaged by the ITR/transgene combination leading to inflammation and cell or synapse destruction.

#### 5.1.3. Decreased Dose

Decreasing the dose of vector required for a sufficient transgene expression is perhaps the most important option as clinical trial data increasingly point to the dose-dependent increase in moderate to severe adverse events. Clinical trials increasingly report adverse events only in the patient cohort injected with the highest dose of rAAV preparation. This is the case in the recent clinical trial for DME (NCT04418427) where patients enrolled in the high-dose cohort presented inflammation and increase of intraocular pressure. In addition, the same observation was made in a phase I/II clinical trial for x-linked retinoschisis, where all patients injected with 1 × 10^11^ vg displayed anterior chamber inflammation around 2 weeks post-administration and vitritis after a month [120]. Even though the inflammation resolved using steroids, only one patient of the 1 × 10^10^ vg and none of the 1 × 10^9^ vg cohorts developed similar adverse events, emphasizing the impact of the vector dose on ocular inflammation [120]. Several preclinical studies or clinical trials reported similar data, with what appears to be a threshold targeting inflammation at above 1 × 10^11^ vg/eye [5,121,122,123,124,125,126,127].

In order to decrease the dose necessary to ensure a proper expression of the transgene, the efficiency of the vector delivery must be increased. As mentioned before, engineered vectors with improved retinal surface transduction and/or that are capable of escaping preexisting AAV immunity are of particular interest. Pavlou and colleagues reported two new variants of AAV2, AAV2.NN and AAV2.GL, which localize in the nucleus with a higher efficiency than native AAV2, 2-fold and 4-fold, respectively [114]. Another strategy could consist of the use of self-complementary AAV (scAAV) vectors that have been described as enhancing transgene expression and could therefore allow lower doses of vector [128]. However, these vectors also increase the innate response to the transgene compared to single-stranded AAV (ssAAV) vectors and may thus not be the most suitable approach [91,129].

### 5.2. At the Patient Level

#### 5.2.1. Immunosuppressive Strategies

Since substantial ocular inflammation has been observed in clinical trials for GT, use of immunosuppressive drugs has become commonplace. However, as recently discussed in a workshop organized by the Fighting Blindness foundation, if immunomodulatory strategies always accompany GT, there is little consensus on optimal molecules and dosing [130].

Glucocorticoids are well-known and long-used immunosuppressive and anti-inflammatory drugs. They act by modulating the gene expression of a number of proteins involved in the inflammatory response. In recent clinical trials, glucocorticoids have been the most widely used anti-inflammatory drugs. Representatives of this family include prednisone, prednisolone, and dexamethasone, which were used pre-operation (for 1 to 3 days) as well as post-surgery (for longer periods of time, up to three weeks), taken orally or applied locally, in multiple clinical trials as well as in conjunction with EMA- and FDA-approved Luxturna [131,132].

Other less common immunosuppressive strategies have been proposed in recent years. It is the case with rituximab (often used as second/third line treatment for uveitis), a chimeric mouse/human monoclonal antibody inducing B-lymphocyte apoptosis and limiting both antibody production and antibody presentation. Another approach consists of proteasome inhibitors, such as carfilzomib and bortezomib, limiting rAAV degradation and antibody presentation; and hydroxychloroquine, inhibiting viral particle recognition by TLR and cGAS [12,133,134,135].

#### 5.2.2. Clinical Trial Criteria

As mentioned before, inflammatory components of the disease of interest may contribute to the immune response following GT, as well as patient history and immunological status. To mitigate the immunogenicity following retinal GT, patients involved in clinical trials should probably be screened more deeply, to establish more stringent exclusion criteria and evaluate risk factors. To date, exclusion criteria for retinal clinical trials vary. For example, one clinical trial for RP due to MERTK mutations (NCT01482195) excludes patients with a preexisting eye condition or who are dependent on the use of an immunosuppressive medication. On the other hand, another trial for RP (NCT04919473) decided to exclude subjects presenting NAbs to AAV2 above 1:1000 and those with active ocular inflammation or a recurrent history of idiopathic or autoimmune-associated uveitis. The criterion may in some cases depend on the nature of the rAAV treatment; however, serology of subjects regarding NAbs is a recurrent criterion for patient exclusion in ocular GT trials as high doses of vectors in patients with preexisting immunity against AAV may leave them at risk. In addition, a clinical history of inflammation and not just of the eye should be systematically taken into account. In the recent clinical trial for DME, all patients showing a strong decrease in intraocular pressure at high dose, had a history of severe vascular disease that potentially led to a pro-inflammatory ocular environment [7]. Risk factors implicated in inflammation might comprise smoking, central adiposity, as well as HLA-B27-positive profile which has already been associated with acute anterior uveitis [136,137]. Thus, several risk factors may be considered before enrolment in a retinal GT clinical trial in order to decrease inflammatory adverse events following rAAV delivery and achieve personalized GT.

## 6. Conclusions and Outlook

As AAV GT has moved from its development phase to clinical application, efforts to “derisk” the vector as much as possible have accelerated. Even minor neuroinflammation has become a concern, in particular with regard to potential bystander effects and danger of losing irreplaceable retinal cells or neurons. In the retina the relatively tiny foveal region critical for visual function compounds this risk. At the bench level, more fundamental research is needed into DNA sensors that work redundantly or in tandem with TLR9, including DHX10, cGAS, and IFI16. For more than a decade, researchers have investigated the link between inflammation and accelerated cellular myddosome formation provoked by vector sequences and vector secondary structure. Such research is only gaining relevance for the clinic as we try to identify the source of inflammatory reactions to AAV. With increased knowledge of these pathways, depending on the composition of the GT product, personalized immunosuppression could be considered. The dose-dependent nature of the immune reactions is likely to spur the ongoing research to find more efficient vectors that can be administered at lower doses. This will allow trials to prioritize the low-dose cohorts as seen recently in a DME GT trial. Another prudent approach would be to screen vectors and limit extraneous sequences that are known to be activating or to prioritize the use of regulatory sequences that have already been proven safe in trials. Of course, such retrospective data are not always available and will be less so for the truly novel AAV vectors to be used for gene editing. For this, in silico algorithmic modelling is likely to be helpful in screening for the “safest” buffer sequences to flank the ITR/transgene cassette. A Bayesian decision-making approach can be incorporated into vector design pipelines as the detailed immune response data from the several CNS/retinal GT trials continue to be published. To help identify these recurring trends in the safety data from phase I/II retinal GT trials, more uniformity in the reporting of adverse immune-related events is necessary. Such guidelines do not necessarily need to be invented and can largely rely on current standards in the diagnostic ophthalmology clinic. For instance, Bouquet et al. reported inflammation according to the Standardization of Uveitis Nomenclature classification [138,139]. By using standardized measures, a direct comparison of responses can be made between retinal GT trials that can be correlated with vector dose, disease-stage, and route of injection. Inflammation can thus be more readily characterized by time-of-onset and response to treatment. Patient screening with exclusion criteria assessed from ethical and scientific perspectives may become even more critical. All these advances and safeguards will facilitate risk mitigation in retinal GT that works with rather than against the immunosuppressive ocular environment.

## Figures and Tables

**Table 2:** RF1 associated with CpG sequences in components of rAAV vectors. (1) CPV Plasmid Bank. (2) Ensembl (−499 to 100 relative to TSS). (3) NCBI database. (4) Ensembl.

Type of Element	Sequence	Size of Sequence	Number of CG Sequences	RF1
AAV sequences	ITR2 left (1)	145	16	11.03%
	ITR2 right (1)	145	16	11.03%
	ITR5 left (1)	166	16	9.64%
	ITR5 right (1)	166	16	9.64%
Promoter	CMV (1)	581	32	5.51%
	CBA (1)	1557	181	11.62%
	CAG (1)	1733	186	10.73%
	hRPE65 (1)	1382	5	0.36%
	hRK (1)	241	10	4.15%
	hRho (1)	848	9	1.06%
	U6 (1)	249	6	2.41%
	GFAP (1)	696	19	2.73%
	EF1a (1)	1179	94	7.97%
	hSyn (1)	485	56	11.55%
	SV40 (1)	269	9	3.35%
	hPGK (1)	507	62	12.23%
	HSP (=P1) (2)	600	83	13.83%
	VMD2 (2)	600	8	1.33%
	Rs1 (2)	600	19	3.17%
Transgene	hRPE65 (1)	1602	21	1.31%
	hRDH12 (1)	960	33	3.44%
	SaCas9 (1)	3156	168	5.32%
	hRDH8 (1)	936	43	4.59%
	ChR2 (3)	930	65	6.99%
	BDNF (3)	3985	87	2.18%
	GNAT2 (3)	1515	29	1.91%
	CNTF (3)	1902	27	1.42%
	eGFP (1)	717	60	8.37%
	SpCas9 (1)	4275	254	5.94%
	Rho (3)	1047	52	4.97%
	VEGFA (3)	1239	95	7.67%
	PDE6b (3)	2565	138	5.38%
	RDCVF (3)	639	50	7.82%
	Cas12a (CpfI) (3)	3903	33	0.85%
	RPE1 (3)	717	29	4.04%
	Mertk (3)	3000	77	2.57%
	RPGR (3)	2448	29	1.18%
	sFLT1 (3)	147	1	0.68%
	Rs1 (3)	675	27	4.00%
	ND4 (4)	1378	31	2.25%
Suppl. elements	IRES (1)	587	29	4.94%
	WPRE (1)	592	37	6.25%
	SV40 PolyA (1)	131	0	0.00%
